# Do associated proximal fibula fractures help predict the severity of tibial plateau fractures?

**DOI:** 10.1007/s00590-023-03513-1

**Published:** 2023-03-14

**Authors:** Duncan B. Mackie, Brendon C. Mitchell, Matthew Y. Siow, Keenan M. Onodera, Garrett K. Berger, William T. Kent

**Affiliations:** 1 Anne and Robert H. Lurie Children’s Hospital of Chicago, Chicago, USA; 2 Department of Orthopedic Surgery, University of California, 200 West Arbor Drive, San Diego, CA 92103 USA

**Keywords:** Tibial plateau fracture, Proximal fibula fracture, Meniscus tear, Bicondylar tibial plateau fracture

## Abstract

**Purpose:**

Proximal fibula fractures are often associated with tibial plateau fractures, but their relationship is poorly characterized. The purpose of this study was to better define the relationship between tibial plateau injury severity and presence of associated soft tissue injuries.

**Methods:**

A retrospective review was performed on all operatively treated tibial plateau fractures at a Level 1 trauma center over a 5-year period. Patient demographics, injury radiographs, CT scans, operative reports and follow-up were reviewed.

**Results:**

Queried tibial plateau fractures from 2014 to 2019 totaled 217 fractures in 215 patients. Fifty-two percent were classified as AO/OTA 41B and 48% were AO/OTA 41C. Thirty-nine percent had an associated proximal fibula fracture. The presence of a proximal fibula fracture had significant correlation with AO/OTA 41C fractures, as compared with AO/OTA 41B fractures (chi-square, *p* < 0.001). Of the patients with a lateral split depression type tibial plateau fracture, the presence of a proximal fibula fracture was associated with more articular comminution, measured by number of articular fragments (mean = 4.0 vs. 2.9 articular fragments, *p* = 0.004). There was also a higher rate of meniscal injury in patients with proximal fibula fractures (37% vs. 20%, *p* = 0.003).

**Conclusions:**

There was a significant relationship between the higher energy tibial plateau fracture type (AO/OTA 41C) and the presence of an associated proximal fibula fracture. The presence of a proximal fibula fracture with a tibial plateau fracture is an indicator of a higher energy injury and a higher likelihood of meniscal injury.

## Introduction

Tibial plateau fractures are frequently encountered injuries, with an estimated incidence of 10/100,000 annual hospital admissions, comprising 1–2% of all fractures [6, 12]. These often complex intra-articular fractures can be managed both operatively and nonoperatively, with open reduction and internal fixation (ORIF) being the preferred treatment for displaced or unstable fractures [15]. Associated soft tissue injuries are often encountered when treating these injuries including meniscal tears and ligamentous injuries of the knee [3, 10].

The implications of an associated proximal fibula fracture in the setting of tibial plateau fractures has been examined in several reports [1, 5, 12, 14, 16]; however, there is a paucity of the literature describing their relationship to fracture classification and injury characteristics. This study aims to enhance our understanding of tibial plateau fractures and their relationship to proximal fibula fractures and meniscal injuries, to improve diagnosis, preoperative planning, and post-operative outcomes in these patients.

## Materials and methods

Following institutional review board approval, a retrospective chart and radiographic review were performed to identify patients who presented to our institution with a tibial plateau fracture between 2014 and 2019. Patients who underwent operative treatment for a tibial plateau fracture were identified through a surgical database, and all diagnosed tibial plateau fractures were confirmed via review of radiographic and computed tomography (CT) imaging. Demographic information, injury radiographs, CT scans, intraoperative findings, and post-operative follow-up data were then compiled for all patients. All confirmed tibial plateau fractures were classified according to the AO/OTA classification system. Patients who were less than 18 years of age or whose fractures were classified as AO/OTA 41A were excluded from the study. A total of 217 tibial plateau fractures in 215 patients met inclusion criteria.

**Fig. 1:**
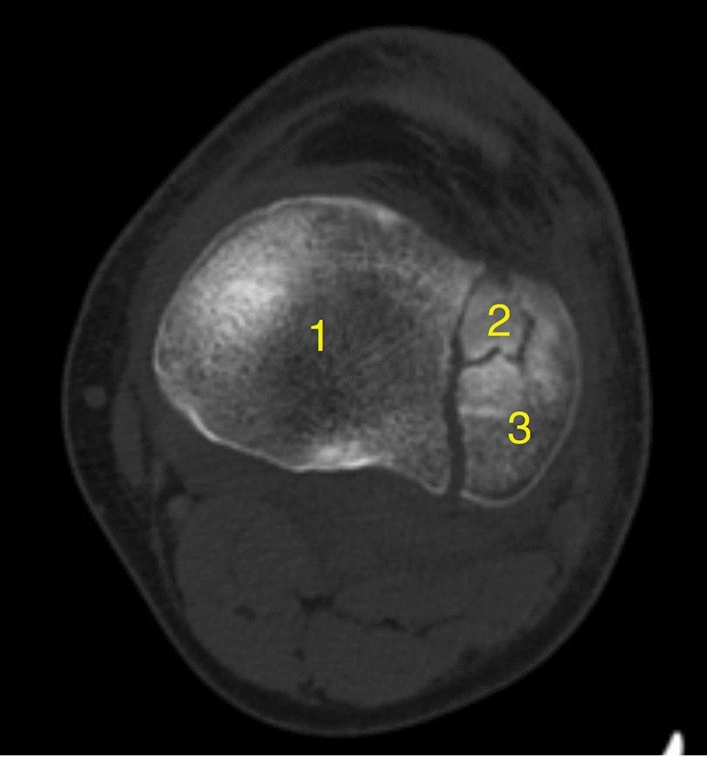
Axial CT of the tibial plateau demonstrating the measurement of the number of articular fragments

**Fig. 2:**
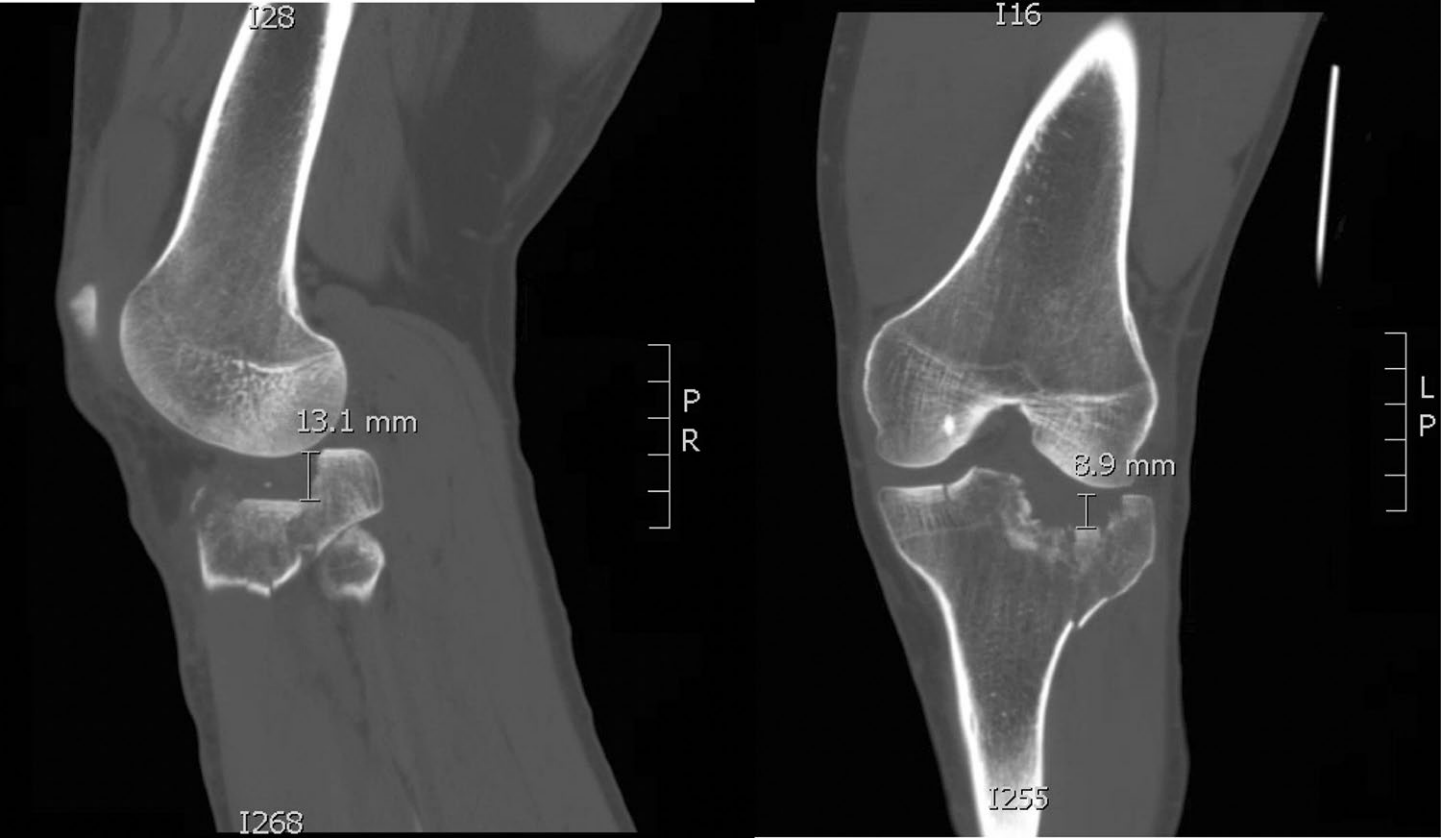
Sagittal and Coronal CT of the knee demonstrating articular depression measured on both views with 13.1 mm of depression on the sagittal, 8.9 mm depression on coronal imaging

To determine the number of fracture fragments associated with each tibial plateau fracture, axial CT scans of each affected extremity were examined. Each discrete fracture fragment was counted and compiled to determine the total number of fragments associated with each tibial plateau fracture (Fig. 1). The degree of joint depression (mm) for each fracture was determined by measuring the fracture fragment with the greatest amount of depression (Fig. 2). Patients with concurrent proximal fibula fractures, defined as AO/OTA 4F1 fracture subtypes, and meniscal tears were identified through review of imaging and operative reports. Patients who were found to have sustained either an isolated mid-shaft or distal fibula fracture were not included in our analysis of the proximal fibula fractures.

### Statistical analysis

Patient demographic data were summarized using mean (standard deviation) for continuous variables and number (percentage) for categorical variables. Independent samples *t* tests were used to compare means, and chi-square tests were used for categorical variables. All analysis was performed using SPSS statistical software, and a* p* value less than 0.05 was considered statistically significant.

## Results

### Cohort and demographics

Two hundred and seventeen tibial plateau fractures in 215 patients (2 patients had bilateral fractures) were included in the study. The mean age was 51.0 years (SD ± 15.1 years) and mean BMI was 27.7 (SD ± 6.2). The study cohort was 34% females (73 patients) and 66% males (142 patients). The average Charlson Comorbidity Index across the study group was 1.9 (SD ± 2.5, Range 0–15). One-hundred and fourteen fractures (52%) in 112 patients were AO/OTA 41B type and 103 fractures (48%) in 103 patients were type AO/OTA 41C. Eighty-five patients (39%) had an associated proximal fibula fracture. The most common mechanism of injury among patients was a fall from height (39%) followed by pedestrian vs. motor vehicle (18%), motorcycle collision (12%), motor vehicle collision, (11%) and other trauma (20%) making up the remainder of the inciting injuries. Twelve patients (5.6%) presented with acute compartment syndrome (ACS), nine of which had fractures classified as AO/OTA 41C (all of which were also Schatzker type VI) and three of which were classified as AO/OTA 41B (all of which were also Schatzker type II). Seventy-five patients (35%) underwent a staged approach with initial placement of a knee spanning external fixator and later definitive fixation when soft tissues were amendable. Demographic data are summarized in Table 1.

**Table 1:** Patient Characteristics

Characteristic	Number of patients (%)
Age (years)	51.0 (± 15.1)^ϯ^
*Gender*	
Male	142 (66%)
Female	73 (34%)
*Tibial plateau fracture classification*	
AO/OTA 41.B	114 (52%)
AO/OTA 41.C	103 (48%)
Patients with concurrent proximal fibula fracture	85 (39%)
*Mechanism of injury*	
Fall from height	84 (39%)
Pedestrian vs. motor vehicle	39 (18%)
Motorcycle accident	26 (12%)
Motor vehicle accident	24 (11%)
Other trauma	42 (20%)
ACS	12 (5.6%)
Staged external fixation prior to ORIF	75 (35%)

*ACS* acute compartment syndrome; *ORIF* open reduction and internal fixation

ϯ = Mean (Standard deviation)

### Proximal fibula fractures convey injury severity

The presence of a proximal fibula fracture was more likely to be seen in AO/OTA 41C fractures, as compared with AO/OTA 41B fractures (56% in the AO/OTA 41C group vs. 25% in the AO/OTA 41B group; *p* < 0.001). A concurrent proximal fibula fracture was associated with more articular comminution at the tibial plateau, although the difference was not statistically significant (4.3 vs. 3.7 fragments, *p* = 0.083). The presence of a proximal fibula fracture was also associated with a higher likelihood of meniscal tear (37% vs. 20%, *p* = 0.003) but not with the overall amount of joint depression (8.5 mm vs. 8.9 mm, *p* = 0.749) or ACS (6.3% vs. 5.2%, *p* = 0.478). Proximal fibula fractures alone were not associated with articular comminution. However, of patients who displayed a lateral split depression type plateau fracture (AO/OTA 41B3)—the most common fracture type in our cohort—the presence of a proximal fibula fracture was associated with more significant articular comminution (4.0 vs 2.9 fragments, *p* = 0.004) (Table 2).

**Table 2:** Summary of results

Variable	Presence of associated fracture	Absence of associated fracture	*p* value
*Effect of proximal fibula fractures on injury severity*	85 (39%)	132 (61%)	
AO/OTA 41.B	28 (25%)	86 (75%)	< 0.001*
AO/OTA 41.C	58 (56%)	45 (44%)	
Comminution (# of fragments)	4.3	3.7	0.083
With lateral split depression type (# fragments)	4.0	2.9	0.004*
Joint depression (mm)	8.5 mm	8.9 mm	0.749
ACS	5 (6.3%)	7 (5.2%)	0.478
Presence of meniscal injury	38 (38%)	23 (19.7%)	0.003*
*Effect of sex on presence of proximal fibula fractures*			
Female sex	27 (37%)	46 (63%)	0.639
Male sex	58 (41%)	86 (59%)	

*ACS* acute compartment syndrome

*Significant with *P* value less than 0.05

## Discussion

Proximal fibula fractures in combination with tibial plateau fractures have been poorly characterized in the literature. While there is a common association between the two, there is a paucity of data describing how the presence of a proximal fibula fracture correlates with associated bony and soft tissue injuries of the tibial plateau. Interestingly, more distally at the ankle joint, these relationships have been well documented with respect to tibial plafond fractures. Barei et al*.* [2] showed that tibial pilon fractures with an associated distal fibula fracture were more severe based on radiographic measures. Additionally, at the distal tibia, the presence of a distal fibula fracture was associated with AO/OTA type C fractures as compared to type B fractures [2]. Luk et al*.* [9] supported this relationship, showing that patients with intact fibulas were more likely to have an AO/OTA B-type fracture, while patients with C-type fractures were more likely to exhibit fibula fractures. The authors concluded that an intact fibula may be predictive of less comminution at the plafond.

In this study we examined the relationship of tibial plateau fracture severity and meniscal injury with the presence of a proximal fibula fracture. Proximal fibula fractures are commonly observed in operatively treated tibial plateau fractures [12]. One study reported an incidence of proximal fibula fractures in patients with tibial plateau fractures at 54% using plain radiography and 61% using CT [8]. Sillat et al*.* [12] demonstrated that detection of an associated proximal fibula fracture using preoperative CT is paramount as these injuries predispose to worse functional scores and are associated with peroneal nerve symptoms.

In a study by Liu et al*.* [8] the authors investigated both the incidence and risk factors associated with combined tibial plateau and proximal fibula fractures. This study classified tibial plateau fractures according to both the Schatzker and 3-column classification systems [8]. The authors assessed for significant associations between the presence of proximal fibula fractures and different types of tibial plateau fractures, as well as the role of both sex and age [8]. They concluded that, in female patients, age increased the likelihood of a complex tibial plateau fracture, particularly those involving the posterolateral articular surface and diaphysis [8]. This study also demonstrated that, in female patients, the presence of a proximal fibula fracture was age dependent [8]. Notably, there was no significant correlation between female sex and proximal fibula fractures.

In our study, proximal fibula fractures were associated with higher energy 41C type plateau fractures. These findings appear to emulate the relationship described with tibial plafond fractures involving the fibula and are consistent with the results of Liu et al*.* [8] where increasing likelihood of a complex tibial plateau fracture coincided with increasing likelihood of a proximal fibular fracture. When further analyzing by specific fracture type, patients with a lateral split depression type (Schatzker II, AO/OTA 41B3) fracture, the presence of a proximal fibula fracture was associated with more significant articular comminution. This finding echoes the results of an investigation by Gardner et al*.* [7] which concluded that these subtypes of tibial plateau fractures demand vigilance in diagnosis as they may be associated with concomitant soft tissue injuries in the setting of joint depression approaching 5 mm. This is important for the planning of both open and arthroscopic interventions for tibial plateau fracture treatment.

Understanding the relationship between the severity of tibial plateau fractures and the degree of surrounding soft tissue injury is critical when it comes to matters such as the timing of surgery, staged external fixation, and planning for incisions [4]. Meniscal injuries have been well described in concurrence with tibial plateau fractures and are important to address at the time of surgery. Stahl et al*.* [13] conducted a study of 602 patients with tibial plateau fractures, of which 179 were identified as having a lateral meniscal tear requiring operative repair. From their results the authors concluded that there may be a correlation between peripheral rim meniscal tears and split depression fractures of the tibial plateau. Another report by Mustonen et al*.* [11] examined the prevalence, type and location of meniscal injuries in the setting of tibial plateau fractures. They found that 36% of patients with a tibial plateau fracture also presented with an unstable meniscal tear [11]. As a result, the authors stress the importance of identifying these injuries in an effort to combine meniscal surgery with fracture fixation and prevent the need for reoperation in these patients [11]. Our study found a significantly higher percentage of meniscal injuries in tibial plateau fracture patients with concomitant proximal fibula fractures. This finding provides another clue for surgeons that a meniscal injury exists. Surgeons should have a high index of suspicion for meniscal injury when a proximal fibula fracture is identified on injury films.

This study is limited by being a single center retrospective review. Another limitation is that this study only included operatively treated tibial plateau fractures which may have only led us to examine the more displaced plateau fractures and their correlation with a proximal fibula fracture. Further studies are needed to identify the relationship between nonoperative tibial plateau fractures, proximal fibula fractures and corresponding meniscal injuries.

This study demonstrates a significant relationship between higher energy tibial plateau fractures (AO/OTA 41C) and the presence of concurrent proximal fibula fractures. Subanalysis within fracture subgroups revealed that proximal fibula fractures, when present with a lateral split depression type tibial plateau fracture, was associated with more articular comminution. The presence of proximal fibula fractures is not only associated with more severe injury to the tibial plateau, but also meniscal tear requiring operative repair. Understanding these relationships may aid in accurate diagnosis of associated injuries with higher energy type fractures, which may influence preoperative planning, approaches, and fixation techniques. Surgeons should be cognizant of a proximal fibula fracture and its correlation with meniscal injury and more significant articular comminution when treating tibial plateau fractures. Further studies are needed to determine the impact of these corresponding injuries on patient outcomes.
